# Cancer Risk Profile of the Levonorgestrel-Releasing Intrauterine Device: A Narrative Review

**DOI:** 10.3390/cancers18152374

**Published:** 2026-07-23

**Authors:** Utku Akgör, Bilal Esat Temiz, Hasan Volkan Ege, Laura Burney Ellis, Sarah Bowden, Maria Kyrgiou, Marc Arbyn, Mario Preti, Kimon Chatzistamatiou, Zoia Razumova, Vesna Kesic, Nicolò Bizzarri, Omar Gassama, Reda Hemida, Nagham Ibraheem, Houssein El Hajj, Deepali Raina, Pierre Collinet, Bernhard Krämer, Murat Gultekin

**Affiliations:** 1 Division of Gynaecological Oncology, Department of Obstetrics and Gynaecology, Faculty of Medicine, Hacettepe University, Ankara 06230, Türkiye; utkuakgor@hacettepe.edu.tr (U.A.); bilalesattemiz@hacettepe.edu.tr (B.E.T.); drvolkanege@hacettepe.edu.tr (H.V.E.); 2 Department of Metabolism, Digestion and Reproduction, Faculty of Medicine, Imperial College London, London W12 0NN, UK; laura.ellis@imperial.ac.uk (L.B.E.); s.bowden@imperial.ac.uk (S.B.); m.kyrgiou@imperial.ac.uk (M.K.); 3 Belgian Cancer Centre, Sciensano, 1050 Brussels, Belgium; marc.arbyn@sciensano.be; 4 Department of Surgical Sciences, University of Turin, 10126 Turin, Italy; mario.preti@unito.it; 5 1st Department of Obstetrics and Gynaecology, School of Medicine, Aristotle University of Thessaloniki, 54124 Thessaloniki, Greece; kimonch@auth.gr; 6 Karolinska Institute, 171 77 Stockholm, Sweden; zoia.razumova@ki.se; 7 Clinic for Obstetrics and Gynaecology, University Clinical Centre of Serbia, Faculty of Medicine, University of Belgrade, 11000 Belgrade, Serbia; vek1@mts.rs; 8 Gynecologic Oncology Unit, Department of Woman, Child and Public Health Sciences, Fondazione Policlinico Universitario Agostino Gemelli IRCCS, 00168 Rome, Italy; nicolo.bizzarri@policlinicogemelli.it; 9 Department of Gynaecology and Obstetrics, Faculty of Medicine, Pharmacy and Odonto-Stomatology, Cheikh Anta Diop University (UCAD), Dakar-Fann P.O. Box 5005, Senegal; omar.gassama@ucad.edu.sn; 10 Mansoura Gynecologic Oncology Unit (MGOU), Mansoura 35516, Egypt; reda11@mans.edu.eg; 11 Baghdad Teaching Hospital, Al-Kindi Medical College, University of Baghdad, Jadirya, Baghdad P.O. Box 47188, Iraq; naghamabrahem1975@gmail.com; 12 Gustave Roussy Cancer Campus, 94805 Villejuif, France; houssein.el-hajj@gustaveroussy.fr; 13 Fortis Memorial Research Institute (FMRI), Gurugram 122002, India; deepali.raina@fortishealthcare.com; 14 Hôpital Privé Le Bois—Ramsay Santé, 59000 Lille, France; pierre.collinet@hotmail.fr; 15 Department for Women’s Health, University Hospital Tübingen, 72076 Tübingen, Germany; bernhard.kraemer@med.uni-tuebingen.de

**Keywords:** levonorgestrel-releasing intrauterine device, hormonal intrauterine system, cancer risk, endometrial cancer, breast cancer, ovarian cancer

## Abstract

**Background/Objectives**: The levonorgestrel-releasing intrauterine device (LNG-IUD) is widely used as a long-acting reversible contraceptive and as a therapeutic option for heavy menstrual bleeding, endometriosis, adenomyosis, and endometrial hyperplasia. Although its predominant effect is local, systemic levonorgestrel exposure has raised questions regarding its potential influence on cancer risk. This review aims to summarise and critically appraise the available evidence on the association between LNG-IUD use and gynaecologic, breast, and selected non-gynaecologic malignancies (including colorectal, lung, pancreatic, gastric, thyroid, skin [melanoma], and haematological cancers). **Methods**: A structured narrative review was developed by a multidisciplinary expert group affiliated with the European Society of Gynaecological Oncology (ESGO) and the European Society for Gynaecological Endoscopy (ESGE). PubMed/MEDLINE, Embase, and Scopus were searched in December 2024 without publication-date restrictions. We critically appraised epidemiological and clinical evidence on LNG-IUD use and breast, endometrial, ovarian, cervical, and selected non-gynaecologic cancer risks, prioritising population-based cohorts, registry-based studies, case–control studies, systematic reviews, and meta-analyses. **Results**: The strongest and most consistent evidence supports a substantial reduction in endometrial cancer risk among LNG-IUD users, with a possible protective association also suggested for ovarian cancer. Available data do not indicate a clear increase in cervical cancer, CIN3+, or invasive cervical disease. In contrast, evidence for breast cancer remains heterogeneous, with some registry-based studies suggesting a modest relative increase and other cohort or case–control studies showing no significant association. Evidence regarding non-gynaecologic cancers remains limited and inconclusive. **Conclusions**: Overall, current evidence suggests that the LNG-IUD has a favourable oncological safety profile, with the strongest evidence supporting a significant reduction in endometrial cancer risk and a potential protective effect against ovarian cancer. There is no evidence pointing to increased risk of cervical cancer. However, results related to breast cancer are mixed, and a slight risk increase cannot be ruled out; thus, counselling should be personalised based on the indication, menopausal status, duration of use, and individual breast cancer risk.

## Simple Summary

The levonorgestrel-releasing intrauterine devices are widely used for contraception and for the treatment of several gynaecologic conditions, including heavy menstrual bleeding, endometriosis, adenomyosis, and endometrial hyperplasia. Because it releases a hormone, its possible relationship with cancer risk has become an important clinical question. This review summarises the available evidence on the association between these devices and different cancers. Current data suggest a clear protective association against endometrial cancer and a possible protective effect against ovarian cancer, while cervical cancer risk does not appear to be increased. Evidence regarding breast cancer remains mixed; some studies report a small increase in risk, whereas others show no significant association. Therefore, counselling should be individualised, especially for women with additional breast cancer risk factors.

## 1. Introduction

The levonorgestrel-releasing intrauterine device (LNG-IUD) is one of the most widely used long-acting reversible contraceptive methods worldwide [1]. Beyond contraception, the LNG-IUD is widely used to treat heavy menstrual bleeding, dysmenorrhoea, endometriosis-associated pelvic pain, and adenomyosis; it provides significant symptom relief and improves quality of life for affected women [2]. In premenopausal and postmenopausal women, the LNG-IUD is also utilised for the treatment of endometrial hyperplasia and, in carefully selected cases, early-stage endometrioid endometrial carcinoma as part of fertility-sparing treatments [3].

Given its widespread use, the measurable systemic absorption of levonorgestrel despite the LNG-IUD’s predominantly local endometrial effects has raised important questions about its potential hormonal influence on cancer development [4]. In parallel, increased awareness of the association between combined oral contraceptives and breast cancer has intensified concerns about the safety of hormonal contraceptives and highlighted the need for evidence to guide counselling on progestin-only drugs or devices, including the LNG-IUD [5,6].

The existing literature on the oncological safety of the LNG-IUD presents a complex and sometimes conflicting landscape. In the context of breast cancer, research remains notably heterogeneous. While some large-scale cohorts and meta-analyses report a 20% to 30% increase in incidence, these findings are often countered by pooled analyses that demonstrate neutral associations. This discrepancy has fuelled a persistent debate over whether observed risk estimates reflect a true biological induction or are a byproduct of methodological differences, such as variations in study design and the extent of adjustment for critical confounders like family history and body mass index (BMI) [7].

In contrast, LNG-IUD use has consistently been associated with a reduced risk of endometrial cancer, likely reflecting the local progestogenic effects of levonorgestrel on the endometrium [6]. Observational studies have suggested a possible protective association between LNG-IUD use and ovarian cancer risk [8]. However, findings remain inconsistent, partly due to differences in adjustment for prior oral contraceptive use and the limited evidence available across histological subtypes of ovarian cancer [6,9]. For cervical cancer, available evidence specific to LNG-IUD exposure does not indicate a clear increase in risk. Nevertheless, interpretation remains challenging due to the frequent pooling of different intrauterine device subtypes in registry-based datasets and potential differences in screening practices among device users [7]. Evidence regarding non-gynaecologic malignancies, including colorectal and lung cancers, remains limited, and currently available studies generally suggest neutral or uncertain associations [4,6].

In this context, a synthesis of the available evidence is necessary to better define the oncological safety profile of the LNG-IUD. This narrative review aims to critically appraise the association between LNG-IUD use and cancer risk—encompassing breast, gynaecological (endometrial, ovarian, and cervical), and non-gynaecological malignancies (including pancreatic, lung, colorectal, gastric, hepatobiliary, thyroid, melanoma, urinary tract, neurological, and haematologic cancers)—to provide a balanced framework for evidence-based clinical counselling.

## 2. Literature Search Strategy

This article presents a structured narrative review to synthesise and critically appraise the available evidence on the association between LNG-IUD and cancer risk. The review was developed by a multidisciplinary group of experts, including gynaecologic oncologists affiliated with the European Society of Gynaecological Oncology (ESGO) Prevention Committee and specialists in gynaecological endoscopy from the European Society for Gynaecological Endoscopy (ESGE), as well as clinical researchers experienced in evidence synthesis. The manuscript was structured in accordance with recommendations for narrative reviews and follows principles outlined in the Scale for the Assessment of Narrative Review Articles (SANRA) framework to enhance methodological transparency and reporting quality [10].

A literature search was conducted in December 2024 using the major biomedical databases PubMed/MEDLINE, Embase, and Scopus, without date restrictions. The search strategy combined terminology related to LNG-IUDs with cancer-related outcomes to maximise sensitivity and minimise the risk of missing relevant studies. The core search strategy included combinations of the following terms: (“levonorgestrel-releasing intrauterine system” OR “levonorgestrel intrauterine device” OR “LNG-IUS” OR “LNG-IUD” OR “hormonal intrauterine device” OR “Mirena”) AND (“cancer” OR “neoplasm” OR “malignancy” OR “carcinoma” OR “tumour” OR “tumour”).

Where appropriate, additional site-specific cancer terms (e.g., “breast cancer,” “endometrial cancer,” “ovarian cancer,” “cervical cancer,” “colorectal cancer,” “lung cancer,” “pancreatic cancer,” “thyroid cancer,” and “melanoma”) were incorporated into the search strategy to identify studies evaluating the association between LNG-IUD use and gynaecologic and non-gynaecologic malignancies. During the study selection process, priority was given to original research with robust study designs, including large population-based cohort studies and case–control studies, as well as relevant systematic reviews and meta-analyses addressing cancer outcomes associated with LNG-IUD use. Case reports and studies with insufficient outcome data or inadequate follow-up were excluded.

To minimise the risk of missing relevant evidence, the reference lists of included articles were manually screened, and citations of key publications were tracked to identify additional relevant studies not captured by the initial database search.

**Figure 1:**
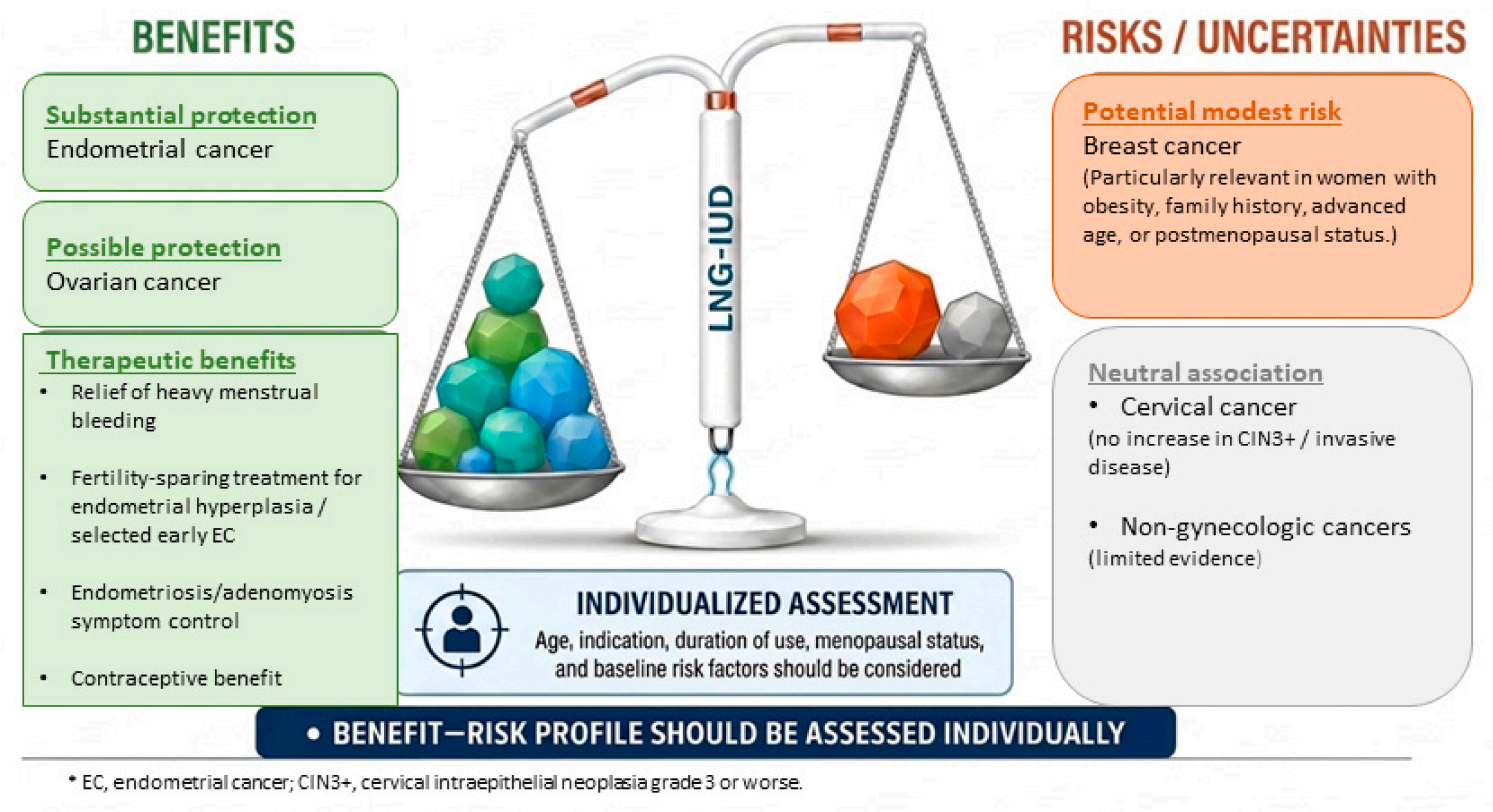
Overall benefit–risk profile of the LNG-IUD in relation to cancer risk. Green indicates benefits or protective associations, orange indicates potential risks or uncertainties, and grey indicates neutral associations or limited evidence.

Generative artificial intelligence (GenAI) was used to assist in generating and refining the graphical layout of Figure 1. GenAI was not used for data collection, data analysis, evidence interpretation, or clinical recommendations. All scientific content, figure labels, and conclusions were critically reviewed and approved by the authors.

## 3. Description and Pharmacokinetics of Levonorgestrel-Releasing Intrauterine Device

Levonorgestrel is a synthetic second-generation progestin with sixfold higher binding affinity for progesterone receptors than natural progesterone [11].

After inserting an LNG-IUD containing 52 mg of levonorgestrel, the initial levonorgestrel release into the uterine cavity is 20 μg per day. A stable plasma level of 150–200 pg/mL is typically established within the first few weeks. These plasma concentrations are lower than those observed with levonorgestrel implants and oral contraceptives [12]. Individual variations in plasma levonorgestrel levels and systemic effects may be due to differences in levonorgestrel binding to sex hormone-binding globulin (SHBG) and metabolic clearance rates [13].

Levonorgestrel primarily exerts its progestogenic effects by binding to progesterone receptors and also interacts with other steroid receptors. It demonstrates a weak agonist effect at glucocorticoid receptors and may exhibit mild androgenic effects due to its moderate affinity for androgen receptors. Additionally, its ability to antagonise mineralocorticoid receptors might help decrease sodium and water retention [14].

The pharmacokinetic profile of the LNG-IUD—characterised by very high local intrauterine levonorgestrel concentrations, reported to be approximately 200–800 times higher than those achieved with daily oral administration, together with low systemic plasma concentrations of approximately 150–200 pg/mL—provides a biologically plausible mechanistic basis for its organ-specific effects on carcinogenesis [11,12]. Within the uterus, sustained local progestogenic exposure suppresses oestrogen-driven endometrial proliferation, induces glandular atrophy and stromal decidualisation, and promotes endometrial thinning [11]. These changes provide a coherent explanation for the consistently observed reduction in endometrial hyperplasia and cancer risk. In contrast, the low systemic levonorgestrel concentrations result in limited progestogenic exposure in extrauterine hormone-sensitive tissues [12,13], which may partly explain the generally neutral or only modest associations reported for breast and other extrauterine cancers. This marked compartmentalisation of levonorgestrel exposure—high within the endometrium but low in the systemic circulation—provides a useful biological framework for interpreting the differing associations observed across cancer sites.

## 4. Potential Biological Mechanisms Underlying Cancer Relationships

A direct carcinogenic or mutagenic mechanism specific to the LNG-IUD has not been demonstrated. Any potential influence on cancer risk is more likely to reflect tissue-specific hormonal and local biological effects than a generalised carcinogenic action. These effects may differ across organs because the LNG-IUD produces very high local levonorgestrel exposure within the uterus while maintaining substantially lower, yet still measurable, systemic concentrations [11]. The principal biological concern relates to breast tissue because intrauterine administration does not completely eliminate systemic levonorgestrel exposure. Levonorgestrel is detectable in both serum and breast tissue, although breast tissue concentrations are substantially lower than those observed with oral levonorgestrel-containing contraceptives [15]. Experimental models suggest that androgenic progestins, including levonorgestrel, may stimulate the proliferation of hormone-responsive breast epithelial cells through androgen receptor–related signalling [16]. Therefore, if the modest epidemiological association with breast cancer is causal, hormonal stimulation of pre-existing susceptible epithelial cell populations may be more biologically plausible than direct mutagenic initiation. However, the clinical relevance of these experimental findings at the low systemic exposure levels achieved with the LNG-IUD remains uncertain. Moreover, limited human breast tissue data have not consistently demonstrated increased cellular proliferation, and causality has not been established [17].

The mechanisms potentially underlying the reported ovarian cancer association remain uncertain. Reduced menstrual bleeding and amenorrhoea may decrease retrograde menstruation, while local foreign-body-related inflammatory responses and anti-oestrogenic effects within the reproductive tract have also been proposed. Partial suppression of ovulation may contribute in some users; however, ovulation frequently continues during LNG-IUD use, making this an incomplete explanation. Overall, these mechanisms do not establish a protective causal effect [18].

For cervical cancer, device-related local inflammation and altered epithelial immunosurveillance, including possible changes in Langerhans cell activity, have been proposed [18]. Conversely, hormonal modulation of innate antiviral factors could theoretically influence HPV persistence [19]. Given these potentially opposing effects and the limited LNG-IUD-specific evidence, no direct mechanism linking LNG-IUD use to either cervical carcinogenesis or cervical cancer prevention has been established. Mechanistic evidence for non-gynaecologic cancers is even more limited, and no consistent causal pathway has been identified.

## 5. Advantages and Therapeutic Indications of Intrauterine Devices

Progesterone-only hormonal intrauterine devices have become some of the most successful methods for preventing pregnancy due to their high efficacy, minimal side effects, and patient satisfaction. LNG-IUDs are particularly effective as a contraceptive option for women unable to use oestrogen-based methods [20].

LNG-IUDs exert therapeutic effects on heavy menstrual bleeding by acting on the endometrium. This effect has been demonstrated in multiple studies, which have consistently yielded similar results [21]. Comparative studies have shown that LNG-IUDs are more effective than various oral progestins in managing heavy menstrual bleeding [22,23]. In women scheduled for hysterectomy due to heavy menstrual bleeding, the use of an LNG-IUD significantly reduces the need for surgery, with rates reported at 64–80%. However, among groups receiving only medical treatment, the rate of hysterectomy avoidance remained 9–14% [24,25].

The LNG-IUD provides clinical advantages for women with hereditary bleeding disorders by markedly decreasing menstrual blood loss. This decline correlates with improved anaemia markers, with an average increase of 1.4 g/dL in haemoglobin and 19.8 ng/mL in ferritin. Furthermore, its use has been associated with improved quality of life [26,27,28].

Various studies have shown that the LNG-IUD is effective in managing endometriosis-related pelvic pain and secondary dysmenorrhoea, as well as primary dysmenorrhoea [29]. It has also been reported to reduce the risk of symptom recurrence when used after endometriosis surgery [30,31]. In patients with adenomyosis, the device provides therapeutic benefits by relieving pain and controlling excessive menstrual bleeding [32,33].

An additional benefit of LNG-IUD is that it can be used in obese patients without contraindications. The local effects of progestins within the uterus provide effective contraception and protect the endometrium in obese women, all while reducing systemic side effects compared with systemic hormonal contraceptive methods [34].

## 6. Association Between Levonorgestrel-Releasing Intrauterine Device Use and Breast Cancer Risk

Evidence suggesting a modest increase in breast cancer risk has mainly emerged from large registry-based studies. In a nationwide Finnish cohort including 93,843 women, LNG-IUD use was associated with a statistically significant increase in breast cancer incidence compared with the general female population, with a standardised incidence ratio (SIR) of 1.19 (95% CI: 1.13–1.25). The association appeared more pronounced among long-term users, particularly those who had used the LNG-IUD for more than five years (SIR 1.40; 95% CI: 1.24–1.57) [6]. A subsequent histology-specific analysis from the same cohort supported this finding and reported a stronger association for lobular carcinoma (SIR 1.73; 95% CI: 1.37–2.15) [4]. Similarly, the nationwide Danish registry study by Mørch et al. [7], which included approximately 1.8 million women aged 15–49 years and more than 20 million person-years of follow-up, reported a modestly increased risk of invasive breast cancer among current or recent users of contemporary hormonal contraception. For LNG-IUD users, the relative risk was 1.21 (95% CI: 1.11–1.33), comparable to that observed with other hormonal contraceptive methods. However, as with other registry-based observational studies, residual confounding, differences in screening behaviour, and lack of detailed information on individual risk factors should be considered when interpreting this association [7]. In a Finnish population-based analysis by Heikkinen et al. [35], including 13,265 breast cancer cases, menopausal status appeared to modify the association between LNG-IUD use and breast cancer risk. An increased risk was observed among postmenopausal LNG-IUD users (OR 1.48; 95% CI: 1.10–1.99), whereas no increased risk was found among premenopausal users (OR 0.77; 95% CI: 0.52–1.13) [35]. Importantly, LNG-IUD use in postmenopausal women often differs clinically from contraceptive use in premenopausal women. In this setting, menopausal hormone therapy typically consists of systemic oestrogen therapy; because unopposed oestrogen increases the risk of endometrial hyperplasia and endometrial cancer in women with an intact uterus, a progestogen is required for endometrial protection. The LNG-IUD may provide this progestogenic component by delivering levonorgestrel locally, thereby avoiding the need for systemic progestin therapy. Consequently, the increased breast cancer risk observed among postmenopausal LNG-IUD users may partly reflect concomitant systemic oestrogen exposure within the context of menopausal hormone therapy rather than an independent effect of the LNG-IUD alone [36].

In contrast, several studies have reported neutral associations. In the Norwegian Women and Cancer (NOWAC) cohort, which included 104,318 women, LNG-IUD use was not associated with an increased risk of breast cancer after multivariable adjustment for age, BMI, parity, physical activity, and oral contraceptive use (RR 1.03; 95% CI: 0.91–1.17) [9]. Similarly, an Israeli cohort study including 13,354 LNG-IUD users aged 40–50 years and 27,324 age-matched controls found no overall increase in breast cancer risk, although a small absolute difference was observed among women aged 40–45 years (0.88% vs. 0.69%; *p* = 0.014) [37]. A Finnish post-marketing study of 17,360 LNG-IUD users aged 30–54 years also found no significant difference in breast cancer incidence compared with the general female population across five-year age groups [38]. Furthermore, a retrospective case–control study conducted in Finland and Germany, including 5113 breast cancer cases and 20,452 matched controls, found no significant association between LNG-IUD use and breast cancer risk. Compared with cu-IUD use, ever-use of LNG-IUD was not associated with increased breast cancer risk (adjusted OR 0.99; 95% CI: 0.88–1.12), and no significant increase was observed among current LNG-IUD users either (adjusted OR 0.85; 95% CI: 0.52–1.39) [39].

More recently, the 2024 Swedish nationwide cohort study by Yi et al. [40] reported a modest increase in breast cancer risk among LNG-IUD users (adjusted HR 1.13; 95% CI: 1.10–1.17). The association appeared stronger among women with a family history of breast cancer, in whom concomitant LNG-IUD exposure was associated with a higher risk estimate (HR 2.07; 95% CI: 1.71–2.51), compared with women without a family history (HR 1.09; 95% CI: 1.04–1.15). Age-stratified analyses also suggested slightly higher estimates among postmenopausal women than among premenopausal women. These findings support the need for individualised counselling in women with additional breast cancer risk factors, although residual confounding and limited duration-specific exposure data remain important considerations [40].

Overall, evidence on LNG-IUD use and breast cancer risk remains heterogeneous. While some large registry-based studies suggest a modest increase in risk, other cohort and case–control studies have shown no significant association. The studies that contributed to this evidence base are summarised in Table 1. These inconsistencies likely reflect differences in menopausal status, baseline risk profile, indication for use, duration of exposure, comparator groups, and adjustment for key confounders. Accordingly, LNG-IUD use should not be considered contraindicated in the general population solely based on current breast cancer data; however, individualised counselling is warranted for women with elevated baseline breast cancer risk, particularly those with advanced age, postmenopausal status, obesity, or a family history of breast cancer.

## 7. Association Between Levonorgestrel-Releasing Intrauterine Device Use and Cervical Cancer Risk

Several articles have examined the risk of cervical cancer associated with intrauterine device use [41,42,43]. However, in most cases, the specific type of intrauterine device is not reported. The first review identified four case–control studies investigating the risk of cervical cancer among intrauterine device users and found either a similar risk or even a decreased risk compared with those who did not report using an intrauterine device [44]. A pooled analysis of 26 epidemiological studies reported that intrauterine device use (predominantly copper intrauterine device) substantially reduces the risk of cervical cancer [41], an outcome consistent with a more recent meta-analysis reporting that, among women who have used an intrauterine device, invasive cervical cancer might be one-third less frequent than among those who have not [42].

In a case–control study of women in the Kaiser Permanente Northern California Healthcare System, 1657 intrauterine device users with incident CIN2+ were compared with 7925 intrauterine device users as controls, and LNG-IUD use was not associated with CIN3+. In particular, regarding the occurrence of cervical cancer, the authors calculated a relative risk (RR) of 1.47 (95% CI: 0.71–3.06, *p* = 0.3) [43]. Another cohort study of 789 intrauterine device users, 354 of whom were LNG-IUD users, compared with intrauterine device non-users and those with an abnormal Pap test, reported no increased risk of cervical cancer among the former group [45].

More robust data come from four registry-based cohort studies—the first of which compared cancer risk in women using an LNG-IUD with that of the general population in Finland. The study reported a standardised incidence ratio of 0.90 (95% CI: 0.69–1.15) among women aged 30–49 who purchased an LNG-IUD at least once over 13 years, compared with women who did not purchase such a device [6]. The second study, from Denmark, compared hormonal intrauterine device users, Cu-intrauterine device users, and oral contraceptive users, directly assessing the incidence of CIN3+. The study reported an adjusted RR for CIN3 or cancer of 1.08 (95% CI, 0.94–1.22), indicating no statistically significant difference between users of hormonal intrauterine devices and users of Cu-intrauterine devices. This suggests that hormonal intrauterine device use would also confer no additional cervical cancer risk, given that multiple studies have previously shown a decreased cervical cancer risk with Cu-intrauterine device use [46].

The third cohort study, from Denmark, investigated data on contemporary hormonal contraception and cervical cancer risk in women aged 15 to 49 years from 1995 to 2014. Among the more than 20 million person-years of the total study population, the study found that LNG-IUD users’ RR for the occurrence of cervical cancer, compared to women who had never used hormonal contraceptives, was 0.76 (0.52–1.11) [47]. The last and most recent study examining the effect of LNG-IUD use on cervical cancer risk was conducted in Sweden, among 16,181 women aged 30–49 years old, and found a slightly increased adjusted odds ratio of 1.45 (95% CI: 1.02–2.06) for the occurrence of histologically confirmed CIN2+ [48]. However, this study included only 297 cases of CIN2+ and did not report separately on CIN3 or cervical cancer, so this result cannot be generalised and interpreted as increased cervical cancer risk. The characteristics and principal findings of the available studies are summarised in Table 2.

## 8. Association Between Levonorgestrel-Releasing Intrauterine Device Use and Ovarian Cancer Risk

The effect of Cu intrauterine devices on ovarian cancer has been investigated for a long time, and most studies have reported neutral or protective results [8]. In contrast, early data on LNG-IUDs were limited, still raising concerns about potential risks. However, large-scale studies conducted in recent years have begun to clarify the picture. One of the most comprehensive cohort studies addressing this issue is the Norwegian NOWAC study, which examined the risk of ovarian cancer among LNG-IUD users in a cohort of 104,380 pre- and postmenopausal women, including 9146 LNG-IUD users, totalling 1,305,435 person-years. Results showed a significant reduction in ovarian cancer risk among LNG-IUD users, with an incidence rate of 16.7 per 100,000 person-years compared with 38.1 among non-users. The age-adjusted relative risk (RR) was 0.49 (95% CI: 0.30, 0.82), and the multivariable-adjusted RR was 0.53 (95% CI: 0.32, 0.88) after adjusting for confounders, including age, contraceptive use, parity, and menopausal status. Although duration-response analysis was limited by case numbers, most ovarian cancer cases among users occurred within the first seven years of use [9].

Soini et al. provided additional insights from two studies involving premenopausal women, in which the use of an LNG-IUD was associated with a lower incidence of ovarian cancer, with an SIR of 0.60 in their first study, which encompassed 855,324 person-years [6]. Their second study, which covered one million person-years, examined the impact of the device on the risk of various histological types of ovarian and primary fallopian tube cancers. The findings were consistent with those of the initial study, with an SIR of 0.59. The protective effect was observed across different histological subtypes, particularly mucinous (SIR 0.49) and serous (SIR 0.75) carcinomas. Conversely, the risk of fallopian tube cancer did not differ significantly between users of the LNG-IUD and the general population [49].

Iversen et al. [50] studied the impact of modern hormonal contraceptives, including levonorgestrel-releasing intrauterine devices, on ovarian cancer risk in a Danish cohort of 1.9 million women (21 million person-years), identifying 1249 ovarian cancer cases. The study found no significant risk reduction in ovarian cancer among users of progestogen-only products, including levonorgestrel-releasing intrauterine devices, in contrast to combined hormonal contraceptives, which showed a protective effect [50].

Balayla et al. [51] pooled nine observational studies and reported a protective association between intrauterine device use and ovarian cancer risk irrespective of device type (OR 0.67; 95% CI 0.60–0.74; *p* < 0.001), with a levonorgestrel-specific subgroup estimate derived from two studies (SIR 0.58; 95% CI 0.47–0.71) [51]. Conversely, D’Alessandro et al. [52] restricted their meta-analysis to levonorgestrel-releasing intrauterine system studies and found no significant risk reduction (OR 0.66; 95% CI 0.41–1.08) [52]. The apparent discrepancy between these two meta-analyses is more likely methodological than biological. Balayla et al. [51] primarily evaluated ever-use of IUDs, with most included studies not distinguishing between copper and levonorgestrel-releasing devices, whereas D’Alessandro et al. [52] specifically focused on LNG-IUD use. Furthermore, adjustment for important confounders, particularly prior oral contraceptive use, was inconsistent across the included studies, and some registry-based cohorts compared LNG-IUD users with the general population rather than an internal unexposed cohort. In addition, D’Alessandro et al. [52] included the large Danish cohort by Iversen et al. [50], which reported no significant protective association for progestogen-only contraceptives, potentially attenuating the pooled estimate. Consequently, although both meta-analyses demonstrated point estimates suggestive of a protective association, the current LNG-IUD-specific evidence remains insufficient to establish a definitive protective effect against ovarian cancer. The available evidence evaluating the association between LNG-IUD use and ovarian cancer risk is summarised in Table 3.

Koskela-Niska et al. [53] assessed the impact of postmenopausal hormone therapies on the risk of ovarian and fallopian tube cancers. They included 3958 ovarian cancer cases and 11,325 controls. They found that the combination of oestradiol and LNG-IUD had no significant effect on ovarian cancer risk, with an odds ratio of 1.02 (95% CI, 0.63–1.66) [53]. In another study, they evaluated the risk of fallopian tube carcinoma and included 360 cases of primary fallopian tube carcinoma and 3442 age-matched controls. They found that use of an LNG-IUD for more than five years was associated with a twofold increase in the risk of primary fallopian tube carcinoma (OR 2.84, 95% CI 1.10–7.38), *p* = 0.032. However, due to the limited number of cases, particularly among long-term users, statistical power was limited [54].

## 9. Association Between Levonorgestrel-Releasing Intrauterine Device Use and Endometrial Cancer Risk

Hormonal imbalances play an essential role in the etiopathogenesis of endometrial cancer, especially oestrogen-dominant hormonal environments with insufficient progesterone. The local progestogenic effect of the LNG-IUD may protect against this mechanism.

The NOWAC study reported a large and statistically significant cancer risk reduction for endometrial cancer with a multivariable-adjusted RR of 0.22 (95% CI: 0.13–0.40), with an especially pronounced risk reduction among women who never used oral contraceptives (RR: 0.08, 95% CI: 0.02–0.34) (p-heterogeneity = 0.18) compared to never-users. Adjustments for age, BMI, physical activity, and reproductive factors were included, and the findings suggest a protective role for the LNG-IUD against endometrial cancer, likely due to its localised progestogenic effects [9]. Soini et al. [6] investigated the use of LNG-IUDs in a cohort of 93,843 Finnish women (855,324 person-years). They found a 54% reduction in the incidence of endometrial adenocarcinoma among users (SIR: 0.46, 95% CI: 0.33–0.64). Among women with multiple LNG-IUD purchases, the risk reduction was even greater (SIR: 0.25, 95% CI: 0.05–0.73) [6].

In a case–control study, Jaakkola et al. [55] assessed the effects of different oestradiol-progestin therapy (EPT) regimens on postmenopausal endometrial cancer risk in Finnish women. This included 7261 endometrial cancer cases and 19,490 age-matched controls. They reported an OR of 0.39 (95% CI: 0.17–0.88), *p* = 0.023, for women using an LNG-IUD for less than 5 years, with continued reductions observed with longer durations of use, OR: 0.16 (95% CI: 0.37–0.68), *p* = 0.013, for durations between 5 and 10 years. The protective effect persisted among users of continuous oestradiol-progestogen therapy. In contrast, prolonged sequential or long-cycle regimens were associated with an increased risk, underscoring the importance of regimen type and duration. Overall, these studies suggest that the LNG-IUD offers endometrial protection, particularly with continuous progestin exposure [55].

Curtis et al.’s [44] systematic review, which analysed ten studies, reinforced this evidence, showing a 40% lower risk of endometrial cancer among intrauterine device users (pooled OR: 0.6, 95% CI: 0.4–0.7), regardless of the type of intrauterine device. The anti-carcinogenic effect of LNG-IUD is attributed to its influence on endometrial atrophy, which mitigates hyperplasia, a precursor to endometrial cancer [44].

In young patients who want to preserve their fertility, an LNG-IUD is used as an option in the treatment of both endometrial hyperplasia and early-stage endometrial cancer in appropriately selected cases [56]. The highest response rate was observed in women under 45 years of age with a BMI of less than 30 kg/m^2^ (84.6%) [57]. According to the European Society of Gynaecological Oncology (ESGO)–European Society for Medical Oncology (ESMO)–European Society of Gynaecological Endoscopy (ESGE) joint recommendations, a combined approach consisting of hysteroscopic tumour resection followed by oral progestins and/or an LNG-IUD represents the most effective fertility-sparing treatment, achieving the highest complete response and live birth rates compared with other conservative options [3].

## 10. The Association Between Levonorgestrel-Releasing Intrauterine Device Use and Non-Gynaecologic Cancer Risk

There are very few published studies on the incidence of non-gynaecological cancers among users of LNG-IUDs compared with non-users. A Finnish cohort study [6] used registry data from 1994 to 2007 and included women aged 30–49 who were coded as LNG-IUD users for menorrhagia (*n* = 93,843) and compared cancer incidence in this cohort with that in the general population. They reported data for several non-gynaecological cancers, including lung and colon cancers, tentatively associated with sex hormones, such as melanoma and other cancers [58]. Lung cancer incidence was lower than expected among those with one or more LNG-IUD purchases compared with the general population, with a standardised incidence ratio of 0.68 (95% CI 0.49–0.91). Pancreatic cancer incidence was also reduced among those with one or more purchases of a levonorgestrel-releasing intrauterine device; the SIR was 0.50 (95% CI, 0.28–0.81). Colon and rectal cancer incidence was slightly increased among LNG-IUD users (SIR 1.17), although this finding was not statistically significant (95% CI 0.99–1.36). Similarly, thyroid: SIR 1.09 (95% CI 0.92–1.28) and melanoma of the skin: SIR 1.08 (95% CI 0.90–1.27). As shown in Table 4, the incidence of stomach, liver, gallbladder and biliary tract, vulvar, vaginal, kidney, bladder and urinary tract, and brain and nervous system cancers as well as non-Hodgkin lymphoma, Hodgkin lymphoma, multiple myeloma, and leukaemia, did not differ significantly among LNG-IUD users.

A similar study used registry data to investigate cancer incidence in women in Shanghai, China (*n* = 66,661) [58]. This study compared the incidence of all and site-specific cancers between ‘ever’ and ‘never’ users of different contraceptive methods, including intrauterine devices. However, it was not specified whether these were exclusively levonorgestrel-releasing, and no 7.5-year follow-up was reported. In this study, no individual cancer type showed a statistically significant difference in incidence between ever- and never-users of intrauterine devices. Similarly to the Finnish cohort, these authors found a lower incidence of lung cancer among ever-users of intrauterine devices compared with never-users, with an HR of 0.80. However, this was not statistically significant (95% CI, 0.56–1.16), and a lower incidence of pancreatic cancer was observed, yielding an HR of 0.87 (95% CI, 0.46–1.63) among ever-users of intrauterine devices. Colon cancer and rectal cancer were not statistically significantly higher among intrauterine device users (colon cancer HR 1.01 (95% CI 0.70–1.47); rectal cancer HR 1.27 (95% CI 0.81–1.98). In contrast to Soini et al. [6], this study found non-statistically significant lower rates of thyroid cancer among intrauterine device users: HR 0.64 (95% CI 0.38–1.07). In addition to gynaecological cancers, including breast, liver, gallbladder, and stomach, incidences were reported, and these three cancers were not statistically significantly different in intrauterine device ever-users compared with never-users.

Figure 1 summarises the overall benefit–risk profile of LNG-IUD use in relation to gynaecologic and non-gynaecologic cancer risk, highlighting substantial endometrial protection, possible ovarian protection, neutral cervical cancer findings, limited evidence for non-gynaecologic malignancies, and the need for individualised counselling in women with elevated baseline breast cancer risk.

## 11. Methodological Gaps in Current Epidemiological Data

The available evidence regarding the association between the LNG-IUD and cancer risk is derived almost exclusively from observational studies, including registry-based cohorts, case–control studies, and meta-analyses. Although these studies provide valuable long-term data, they are inherently susceptible to several methodological limitations that should be considered when interpreting the reported associations.

Residual confounding remains one of the principal challenges. Important determinants of cancer risk—including family history, body mass index (BMI), parity, menopausal status, smoking, screening behaviour, and previous oral contraceptive use—are not consistently available or uniformly adjusted for across studies. In particular, differences in adjustment for prior oral contraceptive exposure may partially contribute to the variability observed in ovarian cancer risk estimates. Furthermore, women receiving an LNG-IUD often differ systematically from comparison groups because of the clinical indication for device insertion, introducing the potential for indication and selection bias.

Additional variability arises from differences in exposure and comparator definitions. Several studies evaluated ever-use of any intrauterine device rather than LNG-IUD-specific exposure, while others compared LNG-IUD users with the general population instead of an internal unexposed cohort. Information regarding duration of use, cumulative exposure, timing of removal, and use of the LNG-IUD as part of menopausal hormone therapy is also frequently unavailable. These differences limit direct comparison between studies and may contribute to between-study heterogeneity. Moreover, the relatively short follow-up of many cohorts, in which cancers among users tend to cluster in the early years of use, may cause late-emerging risks to be missed or early protective signals to be overstated.

Outcome assessment also differs considerably across the literature. Cervical studies have variably reported HPV infection, CIN2+, CIN3+, HSIL, or invasive cervical cancer, whereas ovarian cancer studies have not consistently distinguished histological subtypes. In addition, more frequent gynaecological surveillance among LNG-IUD users may increase the detection of premalignant lesions without necessarily influencing the incidence of invasive malignancies.

Future prospective studies should therefore employ standardised definitions of LNG-IUD exposure, collect comprehensive information on major confounding variables—including prior oral contraceptive use—and ensure adequate follow-up to evaluate long-term cancer outcomes. Reporting both relative and absolute risk estimates, together with analyses stratified by cancer type and histological subtype, will provide more robust evidence and improve individualised clinical counselling.

## 12. Conclusions

Current evidence suggests that the LNG-IUD has a generally favourable oncological safety profile. The most consistent data support a substantial reduction in endometrial cancer risk, plausibly attributable to the endometrium’s strong local progestogenic effect. Available evidence also suggests a possible protective association with ovarian cancer, whereas cervical cancer risk appears neutral, with no clear increase in CIN3+ or invasive disease. Evidence regarding non-gynaecologic cancers remains limited and does not currently support a definitive association.

In contrast, findings for breast cancer remain heterogeneous. Some large registry-based studies suggest a modest increase in risk, particularly among women with additional predisposing factors (advanced age, postmenopausal status, obesity, or a family history of breast cancer). In contrast, other cohort and case–control studies have reported no significant increase in breast cancer risk. Therefore, LNG-IUD use should not be considered contraindicated in the general population solely based on current cancer risk data. However, individualised counselling is warranted, particularly in women with elevated baseline breast cancer risk, advanced age, postmenopausal status, obesity, or a family history of breast cancer. Further well-designed prospective studies with detailed information on duration of use, indication, menopausal status, comparator groups, and baseline breast cancer risk factors are needed to better define the long-term oncological safety of the LNG-IUD. Reporting both relative and absolute risks across cancer sites and histological subtypes would further support individualised counselling.

## Figures and Tables

**Table 1:** Characteristics and main findings of studies evaluating the association between LNG-IUD use and breast cancer risk.

Author (References)	Study Population	Study Design	Method of Evaluation	Effect Estimate	Main Finding
Soini et al. [6]	Finnish registry cohort of LNG-IUD users treated for heavy menstrual bleeding; 93,843 LNG-IUD users	Nationwide registry-based cohort	LNG-IUD users compared with the general female population	SIR 1.19; (95% CI: 1.13–1.25); >5 years SIR 1.40; (95% CI: 1.24–1.57)	Modestly increased breast cancer incidence, particularly among long-term users.
Soini et al. [4]	Finnish LNG-IUD users aged 30–49 years; breast cancers identified before age 55	Nationwide registry-based cohort; histology-specific analysis	Histology-specific breast cancer incidence compared with the general female population	Ductal carcinoma: SIR 1.20; (95% CI: 1.14–1.25). Lobular carcinoma: SIR 1.33; (95% CI: 1.20–1.46). Among women with ≥2 LNG-IUD purchases, lobular carcinoma SIR 1.73; (95% CI: 1.37–2.15)	Increased ductal and lobular breast cancer incidence; highest estimate for lobular carcinoma with repeated purchases.
Mørch et al. [7]	Danish nationwide cohort of women aged 15–49 years; approximately 1.8 million women and >20 million person-years	Nationwide prospective cohort	Current/recent hormonal contraception users versus never-users	Progestin-only intrauterine system: RR 1.21; (95% CI: 1.11–1.33) Absolute excess: 13 additional cases/100,000 person-years	Modest relative increase in breast cancer risk; absolute increase was small.
Heikkinen et al. [35]	Finnish population-based dataset including 13,265 breast cancer cases; analyses stratified by menopausal status	Population-based observational analysis	Breast cancer risk stratified by menopausal status	Postmenopausal: OR 1.48; (95% CI: 1.10–1.99); premenopausal: OR 0.77; (95% CI: 0.52–1.13)	Increased risk was observed among postmenopausal users, whereas no increased risk was found among premenopausal users.
Jareid et al./NOWAC [9]	Participants in the Norwegian Women and Cancer Study; 104,318 women, including 9144 LNG-IUD ever-users and 95,174 never-users;	Population-based prospective cohort	Multivariable-adjusted breast cancer risk among LNG-IUD users. Mean follow-up 12.5 years, contributing 1,305,435 person-years	Breast cancer incidence: 275.7 vs. 281.6 per 100,000 person-years among ever-users and never-users, respectively; multivariable-adjusted RR 1.03; 95% CI: 0.91–1.17	LNG-IUD use was not associated with increased breast cancer risk after multivariable adjustment.
Siegelmann-Danieli et al. [37]	13,354 LNG-IUD users aged 40–50 years and 27,324 age-matched controls	Retrospective cohort	Breast cancer incidence in LNG-IUD users versus age-matched controls	Overall not significantly increased; age 40–45 years: 0.88% vs. 0.69%; *p* = 0.014	No overall increase was observed, although a small absolute difference was reported among women aged 40–45 years.
Backman et al. [38]	Finnish post-marketing cohort of LNG-IUD users aged 30–54 years	Post-marketing observational cohort	100,000 woman-years among LNG-IUD users compared with the general Finnish female population across five-year age groups	Incidence rates per 100,000 woman-years for LNG-IUD users vs. general population: 30–34 years, 27.2 vs. 25.5; 35–39 years, 74.0 vs. 49.2; 40–44 years, 120.3 vs. 122.4; 45–49 years, 203.6 vs. 232.5; 50–54 years, 258.5 vs. 272.6	Age-specific breast cancer incidence among LNG-IUD users was comparable to that of the general Finnish population, with no consistent increase across age groups.
Dinger et al. [39]	Finland and Germany; 5113 breast cancer cases and 20,452 matched controls	Retrospective population-based case–control study	Breast cancer risk among LNG-IUD users compared with copper IUD users	Ever-use of LNG-IUD vs. copper IUD: adjusted OR 0.99; (95% CI: 0.88–1.12). Current use at diagnosis: adjusted OR 0.85; (95% CI: 0.52–1.39)	LNG-IUD use was not associated increased breast cancer risk compared with copper IUD use.
Yi et al. [40]	Swedish nationwide register-based cohort of 514,719 LNG-IUD users aged 18–50 years, matched 1:3 with 1,544,157 non-users between July 2005 and December 2018	Nationwide register-based cohort with propensity score matching	Breast cancer risk among LNG-IUD users, including stratified analyses by family history and menopausal status	Overall HR 1.13; (95% CI: 1.10–1.17); family history HR 2.07; (95% CI: 1.71–2.51); no family history HR 1.09; (95% CI: 1.04–1.15)	LNG-IUD use was associated with a modest increase in breast cancer risk, with higher estimates among women with a family history of breast cancer and among postmenopausal women.

**Table 2:** Characteristics and main findings of studies evaluating the association between levonorgestrel-releasing intrauterine device use and cervical cancer risk.

Author (References)	Study Population	Study Design	Method of Evaluation	Effect Estimate	Main Finding
Castellsagué et al. [36]	2205 women with cervical cancer, 2214 matched controls, and 15,272 healthy women from HPV prevalence surveys; pooled data from 26 epidemiological studies	Pooled analysis of case–control studies and HPV prevalence surveys	Ever-use of IUD versus never-use; adjusted for cervical HPV DNA, number of previous Pap smears, and other covariates	Cervical cancer: OR 0.55; 95% CI: 0.42–0.70; *p* < 0.0001. Squamous-cell carcinoma: OR 0.56; 95% CI: 0.43–0.72. Adenocarcinoma/adenosquamous carcinoma: OR 0.46; 95% CI: 0.22–0.97	IUD use might act as a protective cofactor in cervical carcinogenesis.
Cortessis et al. [37]	Meta-analysis of 16 harmonised case–control and cohort studies; IUD type not distinguished in most included studies	Systematic review and meta-analysis	Point and interval estimates for IUD use and cervical cancer risk extracted from original reports and pooled	Random-effects pooled odds ratio for invasive cervical cancer among ever-IUD users versus never-users: OR 0.64 (95% CI, 0.53–0.77).	IUD use was associated with an approximately 36% lower risk of cervical cancer; invasive cervical cancer may be roughly one-third less frequent among ever-users.
Averbach et al. [38]	Kaiser Permanente Northern California; 17,559 women aged 18–49 with incident CIN2+ (including 1657 IUD users) and 87,378 age-matched controls (including 7925 IUD users), 1996–2014	Case–control study	Recent IUD use within 18 months before the index date; LNG-IUD and copper IUD were evaluated separately for CIN2+, CIN3+, and cervical cancer	LNG-IUD: CIN2+ RR 1.18 (95% CI: 1.08–1.30), *p* < 0.001; CIN3+ RR 1.05 (95% CI: 0.91–1.21), *p* = 0.48. Copper IUD: CIN2+ RR 0.88 (95% CI: 0.75–1.04); CIN3+ RR 0.81 (95% CI: 0.64–1.02)	LNG-IUD use was associated with a modest increase in CIN2 (a lesion with a high regression rate) but not CIN3+ or cervical cancer; copper IUD use showed no association with either outcome.
Curtis et al. [39]	Literature review of studies published 1960–September 2006; four case–control studies evaluating cervical cancer risk in IUD users (device type largely unspecified/older devices)	Systematic literature review	Narrative synthesis of four case–control studies assessing IUD use and cervical cancer risk	All four studies reported similar or decreased risk; no pooled quantitative estimate reported	Intrauterine device use was not associated with an increased risk of cervical cancer.
Frega et al. [40]	789 intrauterine contraception users, including 354 LNG-IUD users and 435 Cu-IUD users; additionally, 1491 non-users with abnormal Pap tests were evaluated as controls	Observational follow-up study	Serial cytology (Pap test), colposcopy, HPV-DNA and HPV-mRNA testing compared between current and ever-users of IUC, by device type and duration of use	No formal RR/OR for cervical cancer was reported. Among women with abnormal cytology, Pap-test severity decreased over time in both IUC users and non-users, with no significant group-by-time interaction (*p* = 0.763). Squamous cell carcinoma at last follow-up was observed in 0/105 IUC users versus 3/1491 non-users (*p* = 0.6455).	IUC use was not associated with persistence, progression, or increased severity of squamous intraepithelial lesions, and no increased occurrence of cervical carcinoma was observed.
Soini et al. [41]	Finnish registry cohort of LNG-IUD users treated for heavy menstrual bleeding; 93,843 LNG-IUD users	Nationwide registry-based cohort	Cervical cancer incidence among LNG-IUD users compared with the general Finnish female population (standardised incidence ratio)	SIR for cervical cancer: 0.90 (95% CI, 0.69–1.15)	No increased cervical cancer risk was observed among LNG-IUD users.
Skorstengaard et al. [42]	Danish population-based register cohort of women aged 26–50 years: 60,551 hormone-containing IUD (HIUD) users, 30,303 copper IUD users, and 165,627 oral contraceptive (OC) users, 2008–2011, with 5-year follow-up	Nationwide register-based cohort	Histology and cytology diagnoses were followed for 5 years after baseline; adjusted relative risks were calculated for CIN2 and CIN3+ among HIUD users compared with CIUD and OC users	For CIN3+ among women with normal cytology at baseline: HIUD vs. CIUD, aRR 1.08 (95% CI, 0.94–1.22); HIUD vs. OC, aRR 0.63 (95% CI, 0.57–0.69).	HIUD users had a similar risk of CIN3+ compared with CIUD users and a lower risk compared with OC users. No signal of increased high-grade cervical precancer risk was observed for hormone-containing IUDs.
Iversen et al. [43]	Danish women aged 15–49 years followed from 1995 to 2014; study population contributed more than 20 million person-years	Nationwide prospective cohort	Cervical cancer risk among users of contemporary hormonal contraception, including LNG-IUD users, compared with women who had never used hormonal contraception	RR for cervical cancer among LNG-IUD users versus never-users of hormonal contraception: 0.76 (95% CI, 0.52–1.11).	LNG-IUD use was not associated with an increased risk of cervical cancer.
Jans et al. [44]	Swedish cross-sectional study; 16,181 women aged 30–49 years (297 CIN2+ cases)	Cross-sectional study	Age-adjusted odds ratios for HPV positivity and histological HSIL+ (CIN2+), comparing LNG-IUD, copper-IUD, and hormonal contraceptive users with non-users	LNG-IUD use was associated with higher odds of hrHPV infection, aOR 1.21 (95% CI, 1.04–1.41), and histological HSIL+, aOR 1.45 (95% CI, 1.02–2.06), in age-adjusted models. No significant difference was observed in HPV clearance rates.	LNG-IUD use was associated with higher prevalence of hrHPV infection and histological HSIL+ in this cross-sectional screening cohort. However, the study did not separately evaluate CIN3+ or invasive cervical cancer.

CI, confidence interval; CIN2+, cervical intraepithelial neoplasia grade 2 or higher; CIN3+, cervical intraepithelial neoplasia grade 3 or higher; HPV, human papillomavirus; LNG-IUD, levonorgestrel-releasing intrauterine device; OR, odds ratio; RR, relative risk; SIR, standardised incidence ratio.

**Table 3:** Characteristics and main findings of studies evaluating the association between levonorgestrel-releasing intrauterine device use and ovarian cancer risk.

Author (References)	Study Population	Study Design	Method of Evaluation	Effect Estimate	Main Finding
Soini et al. [6]	Finnish nationwide cohort of LNG-IUD users; 93,843 women and 855,324 person-years	Nationwide registry-based cohort	Ovarian cancer incidence among LNG-IUD users compared with the general female population	Standardised incidence ratio for ovarian cancer among LNG-IUD users versus the general Finnish female population: SIR 0.60 (95% CI, 0.45–0.76).	LNG-IUD use was associated with a reduced incidence of ovarian cancer.
Jareid et al./NOWAC [9]	Norwegian Women and Cancer Study; 104,380 pre- and postmenopausal women, including 9146 LNG-IUD users; 1,305,435 person-years	Population-based prospective cohort	Observed ovarian cancer incidence among LNG-IUD users was compared with expected incidence in the general Finnish female population using standardised incidence ratios	Ovarian cancer incidence: 16.7 vs. 38.1 per 100,000 person-years among LNG-IUD users and non-users, respectively. Age-adjusted RR 0.49 (95% CI, 0.30–0.82); multivariable-adjusted RR 0.53 (95% CI, 0.32–0.88).	LNG-IUD use was associated with a significantly lower ovarian cancer risk. However, duration-response assessment was limited by the small number of ovarian cancer cases among users.
Soini et al. [45]	Finnish registry cohort; approximately 1,000,000 person-years, histology-specific analysis	Nationwide registry-based cohort; histology-specific analysis	Ovarian and primary fallopian tube cancer incidence among LNG-IUD users compared with the general population	Invasive ovarian cancer: SIR 0.59 (95% CI, 0.47–0.73); borderline tumours: SIR 0.76 (95% CI, 0.57–0.99); mucinous: SIR 0.49 (95% CI, 0.24–0.87); endometrioid: SIR 0.55 (95% CI, 0.28–0.98); serous: SIR 0.75 (95% CI, 0.55–0.99); primary fallopian tube carcinoma: SIR 1.22 (95% CI, 0.49–2.50).	LNG-IUD use was associated with reduced ovarian cancer incidence across several histological subtypes, particularly mucinous and serous carcinomas. No significant reduction was observed for fallopian tube cancer.
Iversen et al. [46]	Danish nationwide cohort of 1.9 million women; 21 million person-years; 1249 ovarian cancer cases	Nationwide prospective cohort	Ovarian cancer risk among users of contemporary hormonal contraception, including progestogen-only LNG-IUD	No significant ovarian cancer risk reduction was observed among users of progestogen-only products, including LNG-IUDs.	Combined hormonal contraception was associated with reduced ovarian cancer risk, whereas progestogen-only methods, including LNG-IUDs, did not show a significant protective association.
Balayla et al. [47]	Meta-analysis pooling multiple observational studies of IUD ever-users (all device types, with a levonorgestrel-specific subgroup analysis)	Systematic review and meta-analysis	Pooled odds ratio for ovarian cancer, ever-use vs. never-use of any IUD, with a pre-specified LNG-IUD subgroup analysis	All intrauterine devices: OR 0.67 (95% CI: 0.60–0.74), *p* < 0.0006; LNG-IUD subgroup: SIR 0.58 (95% CI: 0.47–0.71)	Intrauterine device use, including LNG-IUD, was associated with a protective effect against ovarian cancer.
D’Alessandro et al. [48]	Meta-analysis of 3 studies meeting inclusion criteria (from 34,323 records screened); 1687 ovarian cancer events across 20,461,311 person-years	Systematic review and meta-analysis	Pooled odds ratio for ovarian cancer risk among LNG-IUD users vs. never-users	Random-effects pooled odds ratio for ovarian cancer among LNG-IUD users versus non-users: OR 0.66 (95% CI, 0.41–1.08).	The LNG-IUD-specific pooled estimate suggested a possible reduction in ovarian cancer risk, but the association was not statistically significant.
Koskela-Niska et al. [49]	3958 ovarian cancer cases and 11,325 age-matched controls from Finland	Population-based case–control study	Association between postmenopausal hormone therapy regimens, including oestradiol plus LNG-IUD, and ovarian cancer risk	Oestradiol plus LNG-IUD: OR 1.02 (95% CI, 0.63–1.66).	Oestradiol plus LNG-IUD was not associated with ovarian cancer risk. This evidence relates to postmenopausal hormone therapy rather than contraceptive LNG-IUD use.
Koskela-Niska et al. [50]	Finnish case–control study; 360 primary fallopian tube carcinoma cases and 3442 age-matched controls	Population-based case–control study	Association between LNG-IUD use and primary fallopian tube carcinoma risk, including duration of use	LNG-IUD use for >5 years: OR 2.84 (95% CI, 1.10–7.38), *p* = 0.032.	Long-term LNG-IUD use was associated with increased primary fallopian tube carcinoma risk. However, the number of exposed cases was limited, and statistical power was low.

CI, confidence interval; LNG-IUD, levonorgestrel-releasing intrauterine device; NOWAC, Norwegian Women and Cancer Study; OR, odds ratio; RR, relative risk; SIR, standardised incidence ratio.

**Table 4:** Standardised incidence ratios (SIRs) for site-specific cancers in users of the levonorgestrel-releasing intrauterine device (LNG-IUD), based on the Finnish nationwide cohort by Soini et al. (2014). [6].

Cancer Type	Observed Cases	Expected Cases	SIR (95% CI)	Interpretation
Corpus uteri (all types)	56	94.3	0.59 (0.45–0.77)	Decreased risk
Endometrial Adenocarcinoma	37	79.6	0.46 (0.33–0.64)	Decreased risk
Uterine Sarcomas	18	12.5	1.44 (0.86–2.28)	NS *
Ovarian Cancer	59	98.9	0.60 (0.45–0.76)	Decreased risk
Breast Cancer	1542	1292.2	1.19 (1.13–1.25)	Increased risk
Cervical Cancer	60	66.8	0.90 (0.69–1.15)	NS
Pancreatic Cancer	15	30.3	0.50 (0.28–0.81)	Decreased risk
Lung Cancer	43	63.0	0.68 (0.49–0.91)	Decreased risk
Colorectal Cancer	154	131.4	1.17 (0.99–1.36)	NS
Gastric Cancer	45	40.9	1.10 (0.80–1.47)	NS
Liver Cancer	6	8.7	0.69 (0.25–1.50)	NS
Gallbladder and Biliary Tract	7	7.9	0.88 (0.35–1.81)	NS
Skin Melanoma	129	119.5	1.08 (0.90–1.27)	NS
Kidney Cancer	40	41.0	0.98 (0.70–1.32)	NS
Bladder, Ureter, Urethra	12	12.3	0.98 (0.51–1.70)	NS
Brain and Nervous System	175	168.3	1.04 (0.89–1.19)	NS
Thyroid Cancer	138	126.1	1.09 (0.92–1.28)	NS
Non-Hodgkin Lymphoma	81	75.8	1.07 (0.85–1.32)	NS
Hodgkin Lymphoma	13	10.9	1.19 (0.63–2.03)	NS
Multiple Myeloma	11	11.7	0.94 (0.47–1.68)	NS
Leukaemia	34	36.6	0.93 (0.64–1.29)	NS

Note: Data were derived from Soini et al. [6]; the table was independently constructed and reformatted by the authors. * NS: Not significant.

## Data Availability

No new data were created or analysed in this study. Data sharing does not apply to this article.

## Abbreviations

The following abbreviations are used in this manuscript:

BMI	Body Mass Index
CI	Confidence Interval
CIN2+	Cervical Intraepithelial Neoplasia Grade 2 Or Higher
CIN3+	Cervical Intraepithelial Neoplasia Grade 3 Or Higher
Cu-IUD	Copper Intrauterine Device
EC-Cvc	European Commission Initiative On Cervical Cancer
EPT	Estradiol-Progestin Therapy
ESGE	European Society For Gynaecological Endoscopy
ESGO	European Society Of Gynaecological Oncology
ESHRE	European Society Of Human Reproduction And Embryology
ESMO	European Society For Medical Oncology
Genai	Generative Artificial Intelligence
HPV	Human Papillomavirus
HR	Hazard Ratio
IUD	Intrauterine Device
LNG-IUD	Levonorgestrel-Releasing Intrauterine Device
LNG-IUS	Levonorgestrel-Releasing Intrauterine System
MEDLINE	Medical Literature Analysis And Retrieval System Online
NOWAC	Norwegian Women And Cancer Study
OR	Odds Ratio
RR	Relative Risk
SANRA	Scale For The Assessment Of Narrative Review Articles
SHBG	Sex Hormone-Binding Globulin
SIR	Standardised Incidence Ratio
